# Antiangiogenic Effects and Therapeutic Targets of *Azadirachta indica* Leaf Extract in Endothelial Cells

**DOI:** 10.1155/2012/303019

**Published:** 2012-02-22

**Authors:** Saswati Mahapatra, Charles Y. F. Young, Manish Kohli, R. Jeffrey Karnes, Eric W. Klee, Michael W. Holmes, Donald J. Tindall, Krishna Vanaja Donkena

**Affiliations:** ^1^Department of Urology, Mayo Clinic/Foundation, Rochester, MN 55905, USA; ^2^Division of Biomedical Statistics and Informatics, Mayo Clinic/Foundation, Rochester, MN 55905, USA; ^3^Department of Proteomic Core, Mayo Clinic/Foundation, Rochester, MN 55905, USA; ^4^Department of Biochemistry and Molecular Biology, Mayo Clinic/Foundation, Guggenheim 5-01B, 200 First Street SW, Rochester, MN, 55905, USA

## Abstract

*Azadirachta indica* (common name: neem) leaves have been found to possess immunomodulatory, anti-inflammatory and anti-carcinogenic properties. The present study evaluates anti-angiogenic potential of ethanol extract of neem leaves (EENL) in human umbilical vein endothelial cells (HUVECs). Treatment of HUVECs with EENL inhibited VEGF induced angiogenic response *in vitro* and *in vivo*. The *in vitro* proliferation, invasion and migration of HUVECs were suppressed with EENL. Nuclear fragmentation and abnormally small mitochondria with dilated cristae were observed in EENL treated HUVECs by transmission electron microscopy. Genome-wide mRNA expression profiling after treatment with EENL revealed differentially regulated genes. Expression changes of the genes were validated by quantitative real-time polymerase chain reaction. Additionally, increase in the expression of HMOX1, ATF3 and EGR1 proteins were determined by immunoblotting. Analysis of the compounds in the EENL by mass spectrometry suggests the presence of nimbolide, 2′,3′-dehydrosalannol, 6-desacetyl nimbinene and nimolinone. We further confirmed antiproliferative activity of nimbolide and 2′,3′-dehydrosalannol in HUVECs. Our results suggest that EENL by regulating the genes involved in cellular development and cell death functions could control cell proliferation, attenuate the stimulatory effects of VEGF and exert antiangiogenic effects. EENL treatment could have a potential therapeutic role during cancer progression.

## 1. Introduction

Cancer is profoundly influenced by the tumor microenvironment [1], which is a complex and highly dynamic environment, harboring a variety of host-derived cells involved in tumor development and progression, including endothelial cells, fibroblasts, and innate and adaptive immune cells [2]. Tumor-associated endothelial cells form angiogenic vessels by sprouting from existing vessels and recruit bone-marrow-derived endothelial progenitor cells which provide nutrition to support tumor growth. These cells are also the interface between circulating blood cells, tumor cells, and the extracellular matrix and control leukocyte recruitment, tumor cell behavior, and metastasis formation. Under normal conditions the endothelial lining acts as a barrier against both leukocyte trafficking and cancer-cell transmigration [3]. However, inflammation causes cytoskeletal rearrangements in endothelial cells, cancer cells, and leukocytes, potentially priming them for efficient migration [4]. Subtle changes in endothelial cells phenotype could be easily transmitted to the tumors with profound effects on cancer fate. Endothelial cells can regulate diverse aspects of cancer cell function, including proliferation, invasiveness, and metastasis [5].

Angiogenesis is a necessary process for tumor progression and has emerged as a valid therapeutic target for solid malignancies [6]. Effective inhibition of tumor angiogenesis could provide crucial suppression of not only tumor growth but also tumor metastasis; the development of agents inhibiting angiogenic processes has become a matter of focus. The safety of the approved antiangiogenic agents (e.g., bavacizumab, sunitnib, and sorafenib) is of special concern when taking these agents for longer-term adjuvant or maintenance treatments [6]. Plant extracts and natural compounds possess various bioactive phytochemicals, usually targeting multiple signaling pathways, exhibit less toxicity and thus ideal as alternative and complimentary forms of cancer treatments that involve the dysregulation of multiple genes [7]. This has prompted the recent testing for anticancer potential of numerous plant extracts such as those from green tea, grape seed, pomegranate juice, soy, and garlic [8]. Pomegranate extract is an example having potential clinical effects on treating advanced prostate cancer by prolonging PSA doubling time [9]. One promising medicinal plant is neem with an extensive history in the traditional medicine practices of India and southeast Asia [10]. Neem leaves contain multiple compounds such as limonoids, nonterpenoids, phenolics, and flavonoids that may work simultaneously and/or synergistically to target multiple pathways and suppress the cancer growth [11, 12]. Neem has been shown to cause anticancer, antioxidant, wound-healing, and antimicrobial effects [13, 14]. However, there is currently no information reporting the potential for neem extract to inhibit angiogenesis in cancer.

 The current study is designed to evaluate the antiangiogenic effects of EENL. Our data revealed that EENL inhibited *in vitro* and *in vivo* angiogenic responses, proliferation, migration, and invasion of endothelial cells. To better understand the mechanism of action behind the neem leaf antiangiogenic effects, genome-wide differential transcriptomic analysis was performed. Using the results of the analysis, we identified a set of target genes regulated in endothelial cells after treatment with EENL and then validated the mRNA expression changes and measured the encoded protein expression levels. Further, we performed liquid chromatography/time-of-flight mass spectrometry (LC/TOF-MS) analysis to identify the compounds in EENL. The two individual compounds nimbolide and 2′,3′-dehydrosalannol present in EENL were further tested to inhibit proliferation of HUVECs.

## 2. Materials and Methods

### 2.1. Ethanol Extraction of Neem Leaves

Neem tree leaves harvested during the summer season were obtained from Neem Tree Farms (Brandon, FL, USA). Ethanol extract of the neem leaves was prepared as described previously [15]. The effect of the extract on cell viability and gene expression levels described below were assessed to standardize the method of extraction. We obtained consistent results with different lots of the extract.

### 2.2. Cell Line and Cell Culture

 HUVECs derived from single donors and cryopreserved at the end of passage level-1 were purchased from BD biosciences (Bedford, MA, USA). HUVECs were cultured in Biocoat endothelial cell growth medium (BD Bioscience) supplemented with 5 *μ*g of epidermal growth factor (EGF) and 100 mg of endothelial cell growth supplement (ECGS). The cells were incubated in a humidified atmosphere of 5% CO_2_ at 37°C as described previously [16]. The culture medium was changed every other day and cells were harvested using Trypsin/EDTA solution. Soybean trypsin inhibitor was used to inactivate trypsin for subculturing the cells. For all the experiments described below we used HUVECs before passage level-7.

### 2.3. Cell Viability Assay

 HUVECs derived from single donor were seeded into 96-well plates at a density of 3 × 10^3^ per well for 24 hours at 37°C in a 5% CO_2_ atmosphere and treated with EENL (10 to 80.0 *μ*g/mL) or with the vehicle controls (0.00125 to 0.01% DMSO) for 24 hours. All the experiments were conducted with the HUVECs derived from single donor obtained from BD biosciences. In addition, we further tested the viability of HUVECs pooled from multiple donors purchased from Invitrogen (Carlsbad, CA, USA) using the neem compounds. Nimbolide was purchased from BioVision (Mountain View, CA, USA) and 2′,3′-dehydrosalannol was purchased from the Asthagiri Herbal Research Foundation (Channai, Tamil Nadu, India). A 50 mM solution of these compounds was prepared in dimethyl sulfoxide (DMSO) and stored in small aliquots at −20°C. HUVECs were incubated for 24 hours with nimbolide (2 to 12 *μ*M) or 2′,3′-dehydrosalannol (4 to 20 *μ*M) or with the corresponding vehicle controls (0.004 to 0.04% DMSO). Cell viability was then determined by the colorimetric MTS assay using CellTiter 96 AQueous One Solution Proliferation Assay System from Promega (Madison, WI, USA), as previously described [17]. The number of viable cells is directly related to the absorbance of the medium, when read with a spectrophotometer at 490 nm.

### 2.4. Transmission Electron Microscopy (TEM)

 HUVECs were plated in 10 cm plates and after reaching 60–70% confluency, they were treated for 24 hours with 20.0 and 40.0 *μ*g/mL of EENL. Cells were fixed with Trump's fixative (4% paraformaldehyde with 1% glutaraldehyde in phosphate-buffered saline, pH 7.2), scraped from plates and placed into 2% low melting agar. Processing was facilitated with the use of a laboratory microwave oven (Pelco Biowave 3450, Ted Pella, Inc., Redding, CA, USA). Following fixation, cells were stained with 1% osmium tetroxide and 2% uranyl acetate, dehydrated through an ethanol series, and embedded into Embed 812 resin. Following a 24-hour polymerization at 60°C, 0.1 *μ*M ultrathin sections were poststained with lead citrate. Micrographs were acquired using a Technai G^2^12 Transmission Electron Microscope (FEI, Inc., Hillsboro, OR, USA) equipped with a digital CCD camera (Advanced Microscopy Techniques, Danvers, MA, USA).

### 2.5. Cell Migration and Invasion Assays

 The *in vitro *cell migration and invasion assays were performed using FluoroBlock 24-multiwell insert plates (BD Biocoat angiogenesis system). The insert plates were composed of a fluorescence blocking PET membrane coated with human fibronectin for migration and Matrigel for invasion assays. HUVECs were starved for 5 hours in CS-C medium without serum (Sigma-Aldrich, St. Louis, MO, USA), supplemented with 0.1% BSA for migration assay. HUVECs (1 × 10^4^ cells for migration and 0.5 × 10^4^ cells for invasion) suspended in 250 *μ*L of CS-C serum-free medium supplemented with BSA for migration and growth medium (2% serum) for invasion were treated with EENL (10–80 *μ*g/mL) or vehicle control and seeded onto the upper insert compartment of the transwell chamber. The lower chamber was filled with 2% serum containing endothelial cell growth medium supplemented with chemo-attractant (VEGF 100 ng/mL). After incubating for 22 hours the cells were postlabeled with Calcein AM fluorescent dye. Fluorescence of the invaded cells was read at 494/517 nm. Data were expressed as relative migration and invasion cell number compared with the vehicle control of EENL in the presence of VEGF.

### 2.6. Endothelial Cell Tube Formation Assay

 The *in vitro* tube formation assay was performed using Matrigel-coated angiogenesis plates (BD Biocoat angiogenesis system) as described [18]. HUVECs (4 × 10^5^ cells/mL) suspended in Biocoat endothelial cell growth medium (BD biosciences) were preincubated in the presence of various concentrations of EENL (10 to 80 *μ*g/mL) or the vehicle control and VEGF (100 ng/mL). The cells were seeded in a 96-well angiogenesis plate (2 × 10^4^ cells/well) and incubated for 17 hours at 37°C in a 5% CO_2_ incubator. The cells were labeled with Calcein solution at 8 *μ*g/mL, and tube formation was observed using fluorescent microscope Axiovert 200 M with Apotome Module (Carl Zeiss MicroImaging, Inc.). Tube length was measured using Image pro 6.2 software and the data were expressed as relative tube length compared with vehicle control of EENL.

### 2.7. Measurement of Angiogenic Response *In Vivo*

 The experiments involving mice were conducted with the approval of Executive Subcommittee of the Institutional Animal Care and Use Committee of Mayo Clinic (approval ID: A-25209) in compliance with the Association for Assessment and Accreditation of Laboratory Animal Care International's expectations for animal care and use/ethics committees and the investigators strictly followed the National Institutes of Health guidelines for humane treatment of animals. Female athymic *nu/nu* mice, 6-to-8 weeks of age, were obtained from Charles River Laboratories (Wilmington, MA, USA), and housed at the animal care facility as descried previously [16]. After acclimatization for one week the animals were used for the experiment. Angiogenic response *in vivo* was examined with the directed *in vivo *angiogenesis assay kit (Trivigen, Gaithersburg, MD, USA) as described [19]. In brief, semiclosed angioreactors were filled with 20 *μ*L growth factor reduced basement membrane matrix, 180 *μ*g of FGF-2, 60 ng of VEGF, and 2 *μ*g of heparin with or without 100 *μ*g of EENL. The total volume of the mixture loaded in each angioreactor was kept below 22 *μ*L. The angioreactors were incubated at 37°C for one hour to allow gel formation and then implanted subcutaneously into the dorsal flank of the mice. Two angioreactors were implanted on each right and left dorsal flank of mice, respectively. For each group we used 5 mice, each mouse with 4 angioreactors. Group 1 was the positive control without EENL, group 2 was the test with EENL, group 3 was the negative control without VEGF, and group 4 was the positive control for FGF. After 12 days, angioreactors were removed and photographed. To quantify the angiogenic response, basement membrane matrix/vessel complex from each angioreactor was recovered and digested with CellSperse to collect the cells. The single-cell suspension was labeled with fluorescein-conjugated lectin-1 and the fluorescence was measured in a 96-well plate using Tecan Spectrafluor plus microplate reader at 485 nm.

### 2.8. Gene Expression Analysis and Validation

HUVECs were treated with 20.0 and 40.0 *μ*g/mL of EENL or vehicle control for 24 hours. RNA extraction, gene expression using Human Genomic-U133-Plus2 oligonucleotide microarrays (Affymetrix, Santa Clara, CA, USA) and validation of differentially regulated genes was performed by Taqman real-time PCR as described previously [15]. Ingenuity Pathway Analysis (Ingenuity, Mountain View, CA, USA) was used for evaluation of the biological functions of the differentially expressed genes.

### 2.9. Protein Extraction and Western Blotting

HUVECs were plated in 10 cm plates and after reaching 60–70% confluency were treated for 24 hours with 20.0 and 40.0 *μ*g/mL of EENL. Proteins were extracted from cells in modified RIPA buffer and western blotting was performed using primary antibodies against heme oxygenase-1 (HMOX1), activating transcription factor 3 (ATF3), and early growth response-1 (EGR1), (from Abcam Inc., Cambridge, MA, USA) and horseradish peroxidase-conjugated secondary antibodies as described previously [17]. Immunodetection was performed by LumiGLO chemiluminescence detection system (Cell Signaling, Danvers, MA, USA), in line with the manufacturer's instructions. GAPDH was used as loading control.

### 2.10. LC/TOF-MS Analyses

 LC/TOF-MS analysis was performed as described earlier [15] with a change in the mobile phases using mobile phase A containing water with, 0.1% formic acid, and 0.01% sodium acetate and mobile phase B acetonitrile. Separation was achieved by using a gradient from 65% to 90% mobile phase B over 10 minutes then holding at 90% B for 15 minutes. The scan range for data acquisition was 250–1200 *m*/*z* range. The formation of an even electron sodium adduct in the source was utilized to provide increased selectivity for the tetranortriterpenoids [20] present in the EENL.

### 2.11. Statistical Analysis

 The quantitative data of continuous variables were expressed as mean ± SD. Statistical significance was tested by one way analysis of variance. Fifty percent inhibition concentration (IC_50_) values were calculated by Probit regression. Partek Genomics suite 6.4 was used to analyze the genomic data. A *P* value <0.05 was considered statistically significant.

## 3. Results

### 3.1. EENL Impairs Formation of Tubes *In Vitro* and Vascular Structure *In Vivo*

**Figure 1:**
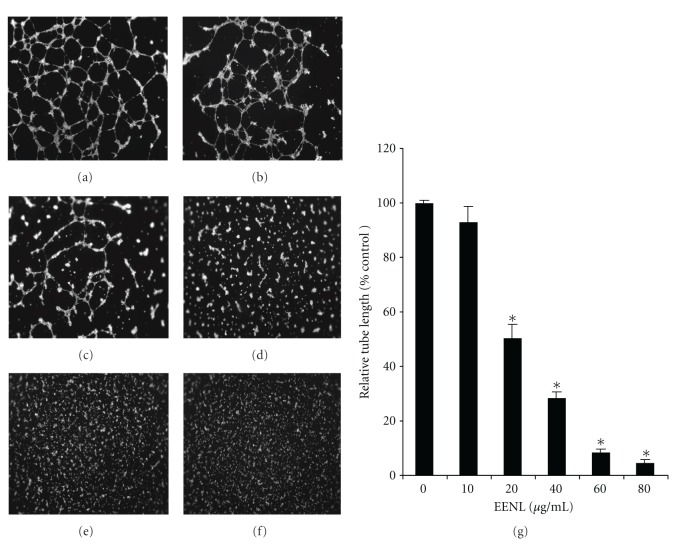
Inhibition of endothelial cell tube formation with EENL treatment. HUVECs were seeded in a Matrigel-coated plate in the presence of 10–80 *μ*g/mL of EENL or the vehicle control and VEGF (100 ng/mL). After incubation for 17 hours the cells were labeled with Calcein solution and tube images were taken at 2.5x magnification. The experiments were repeated twice in triplicate and the representative tube images were shown. (a) Vehicle control treated cells. (b) Cells treated with 10 *μ*g/mL of EENL. (c) Cells treated with 20 *μ*g/mL of EENL. (d) Cells treated with 40 *μ*g/mL of EENL. (e) Cells treated with 60 *μ*g/mL of EENL. (f) Cells treated with 80 *μ*g/mL of EENL. (g) Tube length was measured using Image pro 6.2 software and the mean ± SD of two experiments with each performed in triplicate were shown (*P < 0.05).

**Figure 2:**
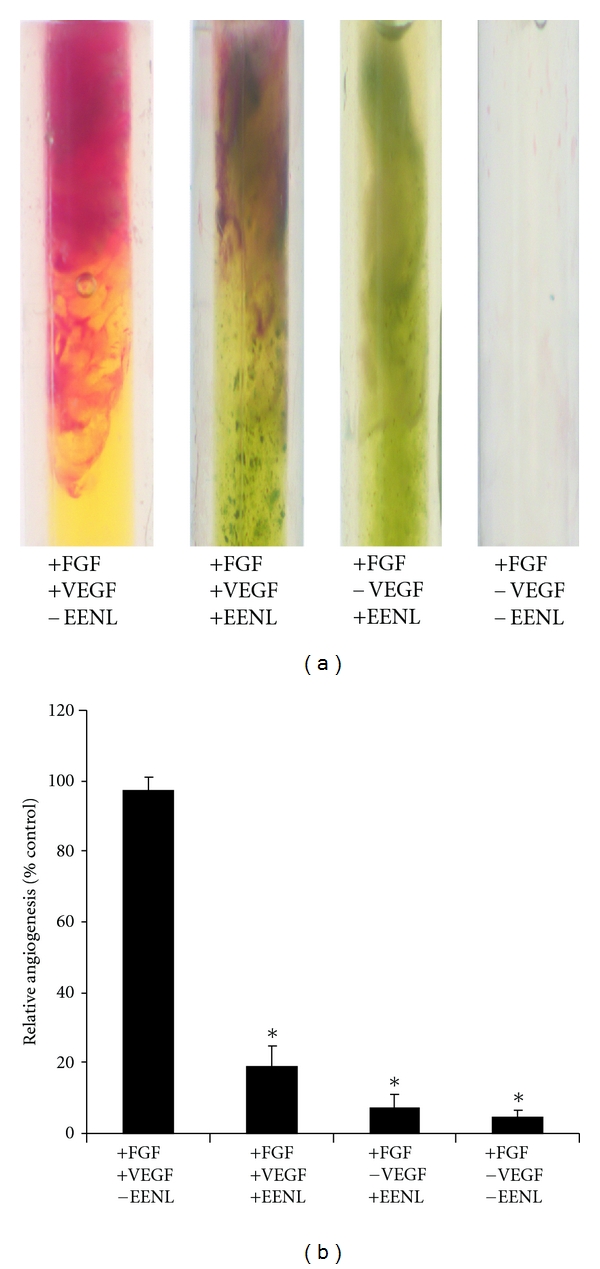
(a) Suppression of vascular growth in the angioreactors with EENL treatment. Angioreactors filled with extracellular matrix containing angiogenic factors (180 *μ*g of FGF-2, 60 ng of VEGF, and 2 *μ*g of heparin) and in presence or absence of 100 *μ*g of EENL were implanted subcutaneously into the dorsal flank of 6-week-old athymic nude mice each. Two angioreactors were implanted on each side of the dorsal flank, a total of four angioreactors per mice and five mice per group were used. After 12 days the angioreactors were removed and photographed. Significant vascular structure is observed in 16 angioreactors of group 1 mice with VEGF. EENL significantly inhibited the VEGF-induced angiogenic response in group 2 mice in a total of 20 angioreactors. Vascular structure was significantly inhibited in all the angioreactors in group 3 and group 4 mice with EENL not containing VEGF and in the negative control without VEGF. (b) Measurement of the angiogenic responses by FITC-lectin staining of the cell pellet recovered from the angioreactors. Data are the mean ± SD from experiments repeated two times (*P < 0.05).

 As tube formation represents one of the late stages of angiogenesis, we evaluated the effect of EENL on *in vitro *tube formation of HUVECs plated on a matrigel substratum. EENL treatment inhibited tube formation and the tube lengths were significantly reduced with 20 to 80 *μ*g/mL of EENL (Figure 1). To substantiate the *in vitro *data, we further studied the effect of EENL on angiogenic response in mice. Semiclosed angioreactors filled with extracellular matrix containing angiogenic factors (heparin, FGF2, and VEGF) with or without EENL were implanted subcutaneously into the dorsal flank of athymic nude mice. The data presented was obtained from 5 mice per group, each mouse implanted with 4 angioreactors from the experiments performed in duplicates. We found apparent vascular structures after 12 days of implantation in the angioreactors of group 1 mice with angiogenic factors in absence of EENL. From the total of 20 angioreactors in five mice from group 1, significant vascular structure was found in 16 angioreactors, whereas vascular structure was not observed in 4 angioreactors that were dislocated from the original position of implantation. The formation of vascular structures induced by the angiogenic factors were significantly inhibited in the presence of EENL in the all the angioreactors of group 2 mice. No vascular structures were observed in angioreactors that were filled with extracellular matrix without angiogenic factors in the presence or absence of EENL in group 3 and group 4 mice, respectively (Figure 2).

### 3.2. EENL Inhibit the Proliferation, Migration, and Invasion of Endothelial Cells *In Vitro*

**Figure 3:**
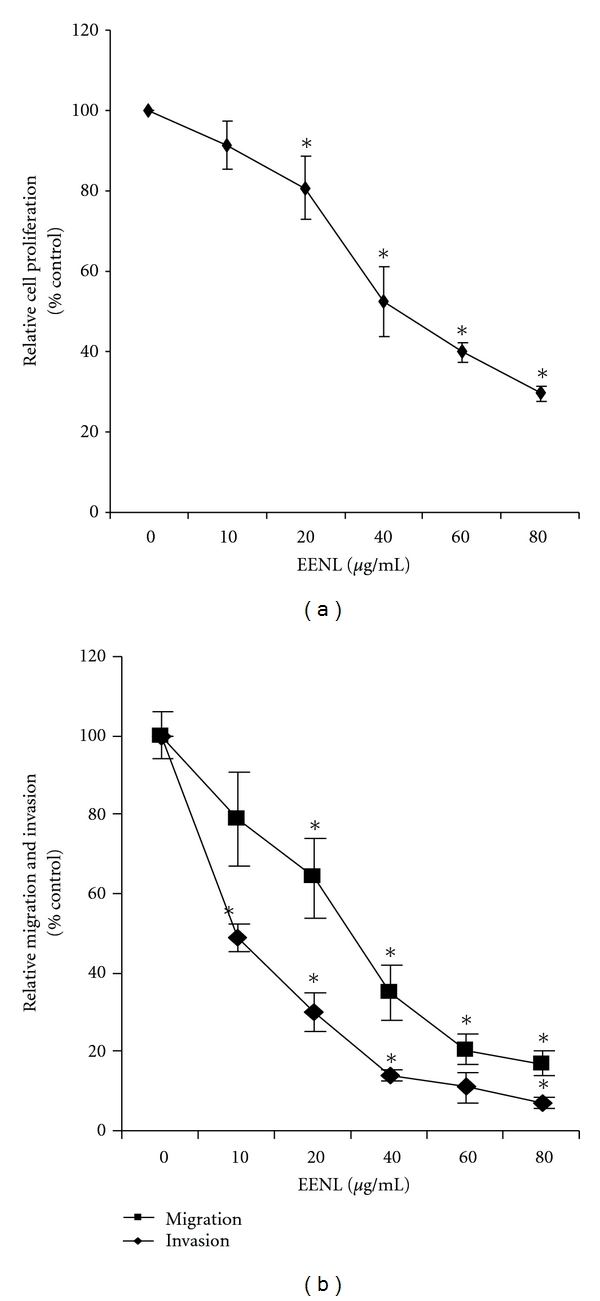
Inhibition of proliferation, migration and invasion of endothelial cells after treatment with EENL. HUVECs were treated for 24 hours with 10.0 to 80.0 *μ*g/mL of EENL or vehicle as control. (a) The antiproliferative effect of EENL was evaluated using the MTS viability assay. (b) Cell migration and invasion were assessed using fibronectin and Matrigel-coated FluoroBlock 24-multiwell insert plates, respectively. Experiments were performed in triplicate and the data were expressed as the mean ± SD of the triplicate determinations of a representative experiment in % cell viability of vehicle-treated cells (100%) (*P < 0.05).

We performed viability assays to analyze the effect of EENL on HUVECs. The antiproliferative activity of EENL was measured by MTS assay. Vehicle treated cells were included as a control. EENL exhibited a dose-dependent inhibition of cell growth with an IC_50_ of 40.0 *μ*g/mL (Figure 3(a)), where IC_50_ is the inhibition concentration at which a 50% inhibition of cell growth is observed at 24 hours of treatment. Endothelial cell migration and invasion, which occur through chemotaxis, are necessary for angiogenesis. We used fibronectin and matrigel Biocoat angiogenesis system to mimic the microenvironment *in vivo* for migration and invasion of the endothelial cells. EENL treatments significantly reduced migration and invasion of HUVECs (Figure 3(b)). The mean inhibitory rates with 10, 20, 40, 60, and 80 *μ*g/mL of EENL were 22%, 37%, 65%, 80% and 84% for migration and 51%, 70%, 86%, 89%, and 93% for invasion, respectively.

### 3.3. EENL Induces Ultrastructural Changes in HUVECs

**Figure 4:**
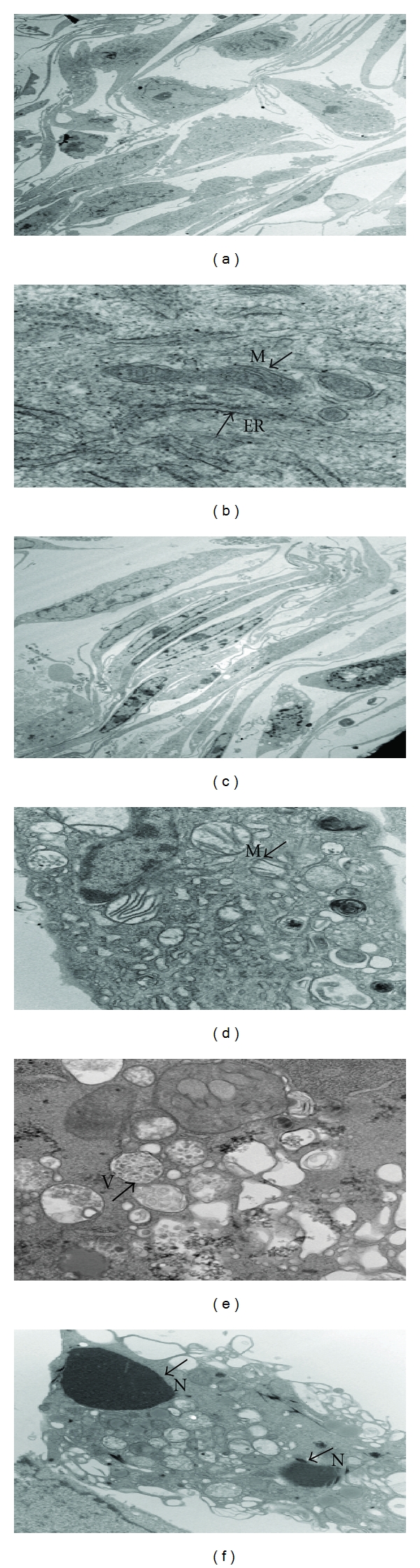
Ultrastructural changes in the HUVECs after treatment with 40 *μ*g/mL of EENL or vehicle for 24 hours observed under TEM. Vehicle-treated cells were used as control. The experiments were repeated in duplicates, and the representative images were shown. (a) Vehicle-treated cells showing morphological structure of HUVECs at 1650x. (b) Mitochondria indicated as *M,* and the endoplasmic reticulum as *ER, *in the vehicle treated cells at 6700x. (c)–(f) Depict cells treated with EENL. (c) Morphological change showing elongated cells at 1650x. (d) Abnormally small round mitochondria with fewer dilated cristae at 2100x. (e) Multivesicular bodies indicated as *V,* in the cells undergoing deterioration at 3000x. (f) Fragmented nuclei indicated as *N,* and the vacuolization in cells at 1100x.

We observed changes in the morphology of EENL-treated cells under light microscope, which prompted us to further examine the ultrastructural changes by TEM. EENL treatment resulted in significant elongation of the cells. Fragmented nuclei, abnormally small mitochondria with dilated fewer cristae, and vacuolization were observed after treatment of HUVECs with 40.0 *μ*g/mL of EENL (Figure 4). These results indicate that EENL induces changes in the cellular function and inhibits cell proliferation.

### 3.4. EENL Regulates Gene Expression Profiles in HUVECs

 To identify the regulation of genes in HUVECs by EENL treatment, we performed high-resolution whole-genome profiling using an Affymetrix microarray mRNA expression platform. The gene expression profiling of HUVECs treated with 20 and 40 *μ*g/mL of EENL for 24 hours showed significant upregulation of 1182 and 3169 genes and downregulation of 1247 and 1893 genes (>2 fold). Using the Ingenuity Pathways Knowledge Base, the independently up- and downregulated genes were mapped for the associated molecular and cellular functions. The most enriched functions in the upregulated genes are cell death and cell morphology, and in the downregulated genes are cell cycle, cellular assembly, and organization, and DNA replication, recombination, and repair, respectively. These functions indicate that EENL may play a significant role in inhibition of cell growth.

### 3.5. EENL Increases the RNA and Protein Expression Levels of HMOX1, ATF3, and EGR1 in HUVECs

**Figure 5:**
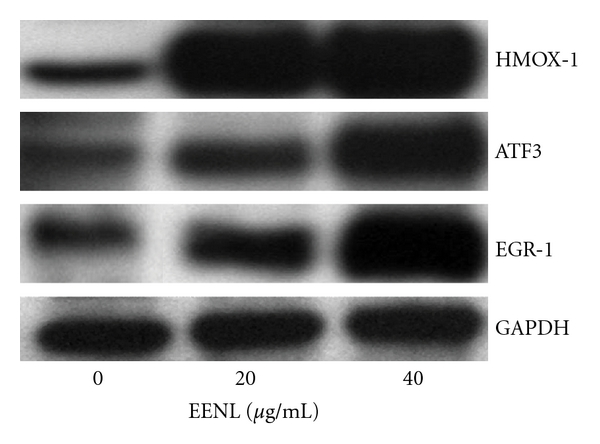
Overexpression of HMOX1, ATF3, and EGR1 in HUVECs after treatment with 20.0 and 40.0 *μ*g/mL of EENL for 24 hours. Protein levels were measured with specific antibodies by western blot analysis; GAPDH was the loading control. Vehicle-treated cells were used as control. The experiments were repeated in triplicates, and the representative blot was shown.

To confirm the alterations of gene expression, we validated the mRNA expression levels of 30 upregulated and 30 downregulated genes for Taqman real-time PCR analysis. The RNA expression levels of the validated up- and downregulated genes treated with 20.0 and 40.0 *μ*g/mL of EENL support the findings obtained from the microarray experiments (Tables 1(a) and 1(b)). We selected 3 significantly upregulated genes to confirm protein expression by western blot analysis. Our results revealed significant increase of the HMOX1, ATF3 and EGR1 protein levels treated with 20 and 40 *μ*g/mL of the EENL (Figure 5) which are consistent with the increase in the RNA expression levels. With the consistency of our results from validation of upregulated genes, we consider that these results could support our microarray data for the validity of the downregulated genes.

### 3.6. Identification of Neem Compounds in the EENL

**Figure 6:**
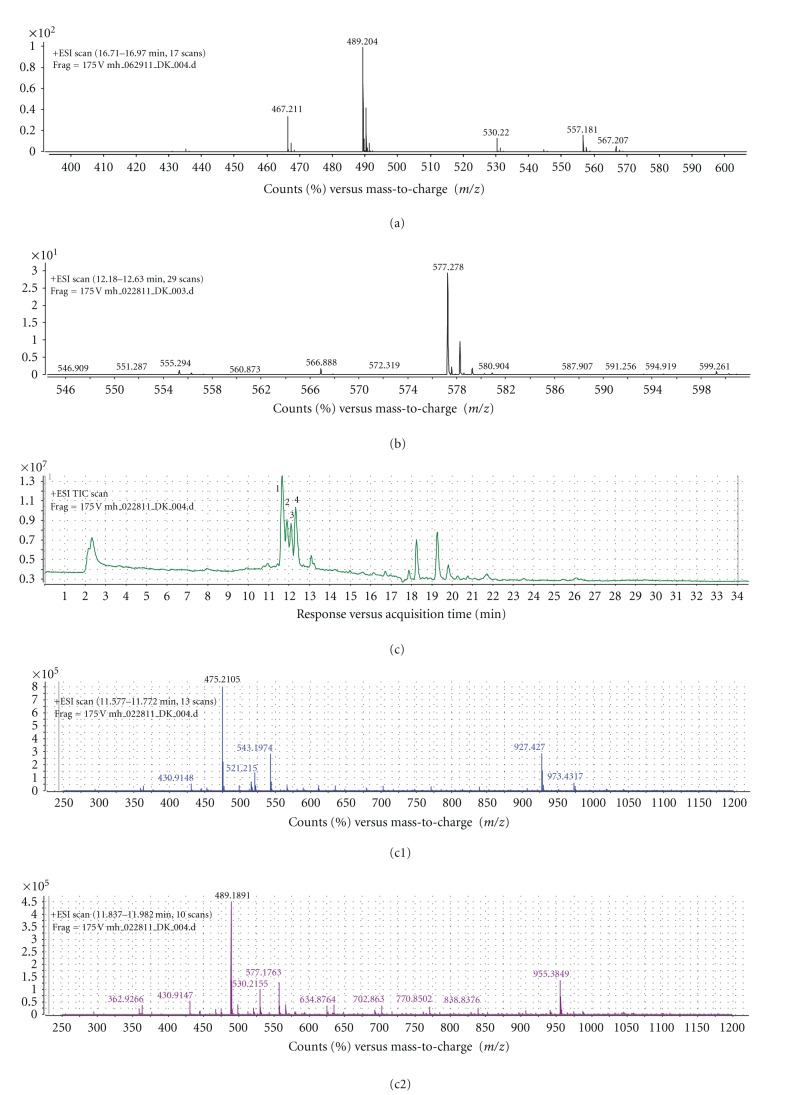

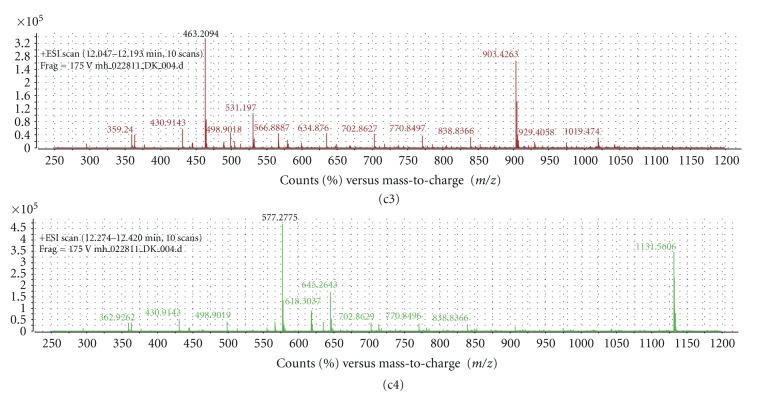
Mass spectrometric analysis of the standards nimbolide and 2′,3′-dehydrosalannol and ethanol extract of neem leaves (EENLs). (a) The mass spectrum of the nimbolide standard depicts the monoisotopic [M + Na]^+^ ion 489.204 *m*/*z* and the [M + H]^+^ ion 467.221 m/z. (b) The mass spectrum of the 2′,3′-dehydrosalannol depicts the monoisotopic [M+Na]^+^ ion 577.2780 *m*/*z*. (c) The total ion chromatogram of EENL. (c1) The mass spectrum of the peak 1 depicts the dominant monoisotopic ion 475.2105 *m*/*z*, at a retention time of 11.68 minutes. Subtracting the mass of sodium (22.9898) from the observed mass results in a mass is suggestive by mass alone as nimolinone. (c2) The mass spectrum of the peak 2 depicts the dominant monoisotopic ion 489.1891 *m*/*z*, at a retention time of 11.91 minutes. After subtracting the sodium mass, this is suggestive by mass and retention time as nimbolide (c3) The mass spectrum of the peak 3 depicts the dominant monoisotopic ion 463.2094 *m*/*z*, at a retention time of 12.12 minutes. Subtracting the mass of sodium from the observed mass results in a mass is suggestive by mass alone as 6-desacetyl nimbinene. (c4) The mass spectrum of the peak 4 depicts the dominant monoisotopic ion 577.2775 *m*/*z*, at a retention time of 12.35 minutes. After subtracting the sodium mass, this is suggestive by mass and retention time as 2′,3′-dehydrosalannol. A comparison of the spectra of the nimbolide and 2′,3′-dehydrosalannol standards to the spectra from the EENL peaks suggests that peaks 2 and 4 are nimbolide and 2′,3′-dehydrosalannol, respectively.

 LC/TOF-MS analysis was performed as described earlier [15] with the modification by including alkali cation sodium acetate to enhance the identification of the potential active compounds in the EENL. Our analysis indicated mass spectral peaks that appear to match the calculated monoisotopic masses of known neem leaf compounds including nimbolide and 2′,3′-dehydrosalannol as standards. The mass spectrum of the peaks includes protonated sodium adducts of the standards. We subtracted the mass of sodium, 22.9898, from each peak. The calculated monoisotopic mass of nimbolide is 466.199 (molecular formula: C_27_H_30_O_7_) and for 2′,3′-dehydrosalannol is 554.2880 (molecular formula: C_32_H_42_O_8_). The observed nimbolide standard showed a mass spectrum with a major peak [M+Na]^+^ at 489.204 m/z and a lesser intensity peak [M+H]^+^ at 467.211 m/z. Subtracting the mass of sodium from the observed 489.204 m/z results in a monoisotopic mass of 466.231, and subtracting the mass of hydrogen from the observed 467.221 m/z results in a monoisotopic mass of 466.213. These adjusted values are in close agreement with the calculated monoisotopic mass for nimbolide. The observed 2′,3′-dehydrosalannol standard showed a mass spectrum with a major peak at 577.2780 m/z. Subtracting the mass of sodium from the observed 577.2780 m/z results in the calculated monoisotopic mass of 554.288. This adjusted value is in close agreement with the calculated monoisotopic mass for the 2′,3′-dehydrosalannol. The total ion chromatogram of the EENL depicts 4 significant peaks, based on the intensity. These peaks were labeled 1, 2, 3, and 4; the associated major ions for the peaks are 475.2105 m/z, 489.1891 m/z, 463.2094 m/z, and 577.2775 m/z, respectively. After subtracting for the mass of a sodium ion, the major adjusted mass for peaks 1, 2, 3, and 4 are 452.2210, 466.199, 440.220, and 554.288, respectively. These adjusted masses from peak 2 and peak 4 are in close agreement with the observed masses of the nimbolide and 2′,3′-dehydrosalannol standard and suggesting that they are compounds of EENL. The calculated monoisotopic mass for the compound nimolinone (C_30_H_44_O_3_) is 452.3290 and 6-desacetyl nimbinene (C_26_H_32_O_6_) is 440.2199. The observed mass spectra for nimolinone and 6-desacetyl nimbinene show major monoisotopic masses of 452.2220 and 440.2195, respectively. The mass measurements for the dominant monoisotopic masses observed for peaks 1 and 3 are in agreement with the calculated monoisotopic masses of nimolinone and 6-deacetyl nimbinene, which suggests their presence in EENL. The EENL, the nimbolide standard, and the 2′,3′-dehydrosalannol standard were analyzed in duplicate representative chromatograms and spectra were shown in Figure 6.

### 3.7. Nimbolide and 2′,3′-Dehydrosalannol Inhibit the Proliferation of HUVECs

**Figure 7:**
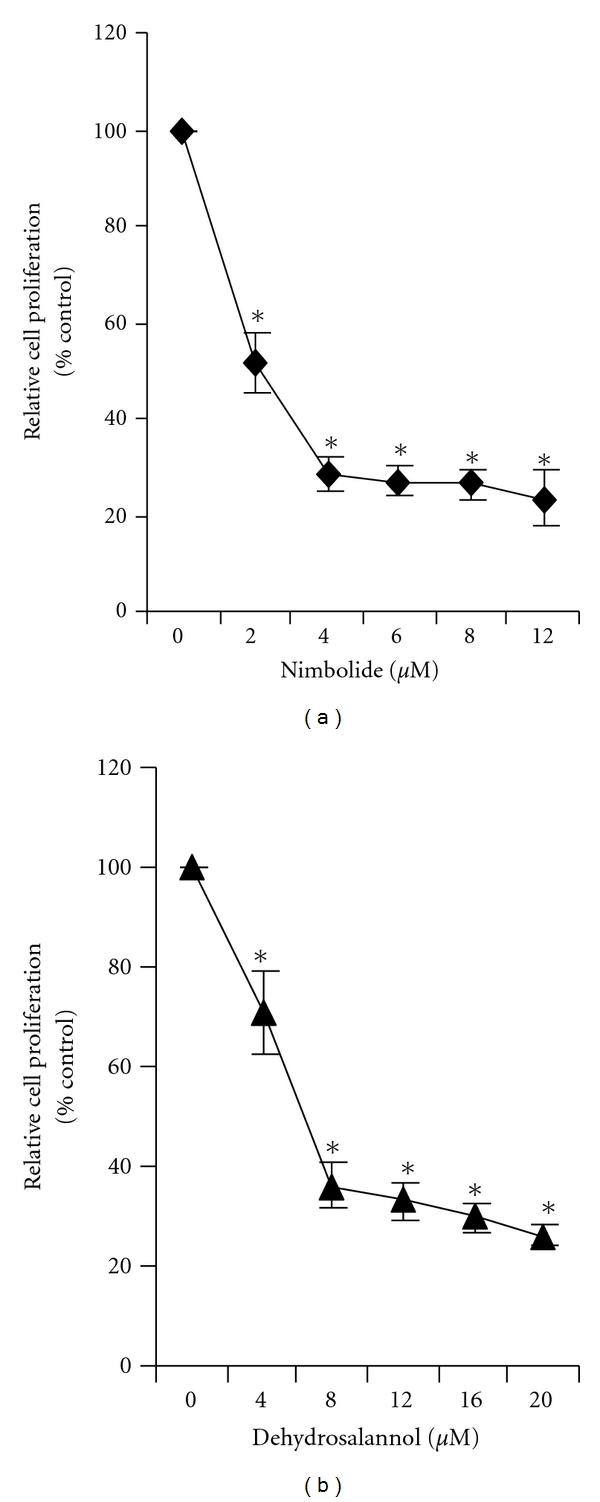
Inhibition of proliferation of endothelial cells after treatment with (a) nimbolide and (b) 2′,3′-dehydrosalannol. HUVECs were treated for 24 hours with nimbolide (2–12 *μ*M) or 2′,3′-dehydrosalannol (4–20 *μ*M) or vehicle as control. The antiproliferative effect of the two compounds was evaluated by using the MTS viability assay. Experiments were performed in triplicate and the data were expressed as the mean ± SD of the triplicate determinations of a representative experiment in % cell viability of vehicle-treated cells (100%) (*P < 0.05).

We further tested the neem compounds to inhibit proliferation of HUVECs pooled from multiple donors. A dose-dependent inhibition of HUVECs growth was observed with an IC_50_ of 2.0 *μ*M for nimbolide and 6.0 *μ*M for 2′,3′-dehydrosalannol after 24 hours of treatment (Figure 7). Our results suggest that the antiproliferative effect of EENL could be a result of the effect of nimbolide and 2′,3′-dehydrosalannol.

## 4. Discussion

 This study is focused on the use of the extract because of combining the effects of the compounds in EENL which could have multitargeted approach for regulation of multiple signaling pathways in cancer progression. Cytotoxic and antitumor effects of neem leaf extracts have been reported in a panel of cancer cell lines [21, 22]. In our previous study, we have demonstrated antiproliferative activity of EENL *in vitro* in prostate cancer cell lines and antitumor activity *in vivo* in prostate cancer xenograft models [15]. In this study we evaluated the effect of EENL on angiogenesis by assessing the tube formation of endothelial cells using a matrigel culture system. We observed that EENL inhibited VEGF-induced *in vitro *tube formation of HUVECs in a dose-dependent manner (Figure 1). Moreover, we have shown that EENL is able to block development of vasculature *in vivo* induced by angiogenic factors which is essential for new vessel development that leads to tumor cell proliferation and invasion (Figure 2). These results suggest that antiangiogenesis activity of EENL is associated with inhibition of VEGF activity and EENL can be recognized as a therapeutic candidate of anticancer drugs.

 We further explored the effect of EENL on proliferation, invasion, and migration of endothelial cells. The extent of HUVECs growth inhibition was measured by MTS assay which was used to determine the number of viable cells in proliferation. EENL treatment significantly inhibited the growth of HUVECs (Figure 3(a)). Furthermore, migration and invasion assays showed that HUVECs treated with EENL showed markedly attenuated migration and invasion in a concentration-dependent manner (Figure 3(b)). Mitochondria are key organelles in conversion of energy, regulation of cellular signaling, and amplification of programmed cell death. A growing body of evidence shows changes in mitochondrial shape are related to its altered metabolic state affecting the regulation of cellular functions [23]. Ultrastructural changes in the HUVECs observed by TME suggest that EENL plays a vital role in altering the cellular functions (Figure 4). Further studies are required to elucidate the mechanism of action of EENL on the inhibition the cell growth.

 In order to identify the molecular targets involved in mediating the effect of EENL in endothelial cells, we used genome-wide gene expression microarray analysis. HUVECs showed a significant upregulation of 1182 genes and downregulation of 1247 genes after treatment with 20.0 *μ*g/mL of EENL, and 3169 upregulated genes and 1893 downregulated genes with 40.0 *μ*g/mL of EENL. We further validated the expression of 30 upregulated and 30 downregulated genes by quantitative real-time PCR. There was a significant upregulation of the genes ALDH3A2, ALOX5, ATF3, CLU, EGF, EGR1, FOXC1, GCLM, HMOX1, JDP2, LY96, PEG3, S100P, and SPRR1A. These genes are associated with kidney failure, renal disease, and urological disease [24]. Significant up-regulation was also confirmed for AKR1B10, AKR1C1, AKR1C2, AKR1C3, CHAC1, CSTA, DDIT3, DMRT1, GPNMB, ID2, LAMP3, SESN2, SPINK1, TRIM16, and TUBA1A. These genes are involved in cellular development and cell death functions [25]. The majority of the downregulated genes, including ANKRD12, ASPM, CDC25A, CDCA4, CENPE, CHEK1, DLGAP5, DPP4, DTL, EBP, E2F8, FBXO5, FOLH1, HIST1H4C, HSP90B1, ITGAV, KIF14, MAD2L1, METAP2, NRIP1, POLA1, PRIM2, SKP2, TOP2A, TOP2B, TPR, and UHRF1, are associated with cell cycle function and cancer development [26, 27]. All these validated upregulated and downregulated genes have been shown to be down-regulated and up-regulated, respectively (Tables 1(a) and 1(b)), in various cancer tissues, as shown in Oncomine microarray data base [28]. This again suggests EENL could induce an inhibitory effect on cancer growth and should be further considered for the prevention and treatment of cancer.

 To correlate gene expression changes with protein levels, we selected 3 upregulated genes HMOX1, ATF3, and EGR1 for western blot analysis. HMOX1, an enzyme degrading heme to carbon monoxide, iron, and biliverdin, has been recognized as playing a crucial role in cellular defense against stressful conditions [29]. Although the expression of HMOX1 is low in most tissues, a large number of plant extracts and pharmacological compounds (e.g., green tea, curcumin, and statins) have been demonstrated to induce HMOX1 [30–32]. Pharmacologic inhibition of HMOX1 induces marked leukocyte infiltration and enhances VEGF-induced angiogenesis [33]. Overexpression of HMOX1 in prostate cancer cells downregulated the MMP9 expression and decreased the invasive potential [34]. The inhibition of invasion and migration of HUVECs following EENL treatment could be the result of a highly significant increase in the RNA and protein expression levels of HMOX1 (Table 1 and Figure 5). Under prolonged expression of HMOX1 the released iron, carbon monoxide, biliverdin, and bilirubin from the HMOX1 activity may attenuate the stimulatory effects of VEGF and exert antiangiogenic effect [35]. The induction of HMOX1 by EENL treatment could be the possible mechanism to attenuate the excess formation of blood vessels in inflammatory angiogenesis of the cancer.

 ATF3 is a novel stress-activated regulator of p53 protein stability and function [36]. It provides the cell with a means of responding to a wide range of environmental insult, thus maintaining DNA integrity and protecting the cell against transformation [37]. ATF3 has been demonstrated to play a role in apoptosis and proliferation, two cellular processes critical for cancer progression [38]. Enforced expression of ATF3 can restore p53 activity and induce apoptosis of cells [39]. EENL treatment increased the expression of ATF3 in HUVECs. Targeting ATF3 expression through EENL could be a promising strategy for cancer therapy. EGR-1 is an immediate early gene that is rapidly and transiently induced by many stimuli, including hypoxia, shear stress, and injury [40]. EGR-1 controls the expression of a wide variety of genes, many of which play a pivotal role in the regulation of cell growth, differentiation, and apoptosis [41]. However, although several studies have shown EGR-1 promotes cancer progression, there is increasing evidence that EGR-1 may also exerts tumor suppression [42, 43]. In leukemia, EGR-1 has been implicated in the apoptosis of myeloma cells via interaction with c-JUN while it behaves as a tumor suppressor against the oncogenes E2F-1 and c-MYC [44]. EGR-1 activation by EGF inhibited MMP9 expression and lymphoma growth [45]. EENL induced significant upregulation of EGR-1 expression in endothelial cells (Table 1 and Figure 5) suggesting that EGR1 might have negative effects on endothelial cells. Further studies are needed to elucidate the role of EGR-1 upregulation on the endothelial cell function.

 Analysis of the compounds in EENL by LC/TOF-MS including sodium acetate in the mobile phase revealed 4 major peaks which suggest 2′,3′-dehydrosalannol, nimbolide, 6-desacetyl nimbinene, and nimolinone as the major compounds in the EENL (Figure 6). The formation of an even electron sodium adduct in the source was utilized to provide increased selectivity for the tetranortriterpenoids. Compared to the formation of the protonated adduct as presented earlier [15], the present data reveals that addition of the basic ion can pick up nimbolide present in the EENL. Further testing revealed that nimbolide and 2′,3′-dehydrosalannol inhibited HUVECs proliferation (Figure 7), suggesting that these compounds contribute to the antiproliferative activity of EENL.

 In conclusion, these findings suggest that EENL containing the compounds nimbolide, 2′,3′-dehydrosalannol, 6-desacetyl nimbinene, and nimolinone inhibits proliferation, migration, invasion and angiogenesis response of HUVECs. We used genome-wide profiling approach to identify the genes associated with multiple biological pathways as potential targets of EENL, and demonstrated the upregulation of the HMOX1, ATF3, and EGR-1 protein expression in HUVECs. Over-stimulation of HMOX1 production could be one of the contributing factor for inhibition of VEGF induced angiogenesis. Our study indicates the importance for further validation of the antiangiogenic potential in preclinical models and in clinical trials, for successful translation of nontoxic neem treatment into the clinic to prevent tumor progression.

## Figures and Tables

**Table 1a:** (a)

Gene	Fold upregulation (20 *μ*g/mL)	Fold upregulation (40 *μ*g/mL)	Assay ID	Function
AKR1B10	17.68 ± 3.25	48.31 ± 8.68	Hs00252524_m1	Aldo-keto reductase activity
AKR1C1	6.70 ± 1.51	23.13 ± 5.43	Hs00413886_m1	Oxidoredutase activity
AKR1C2	2.95 ± 0.55	23.31 ± 5.07	Hs00912742_m1	Oxidoredutase activity
AKR1C3	2.73 ± 0.87	4.47 ± 2.67	Hs00366267_m1	Oxidoredutase activity
ALDH3A2	2.01 ± 1.33	3.97 ± 2.96	Hs00166066_m1	Aldehyde dehydrogenase
ALOX5	7.74 ± 2.50	1.97 ± 1.42	Hs01095330_m1	Lipoxygenase activity
ATF3	86.64 ± 12.44	277.09 ± 16.31	Hs00231069_m1	Transcription factor
CHAC1	54.21 ± 4.2	156.95 ± 14.98	Hs00225520_m1	Protein binding
CLU	2.53 ± 1.04	4.47 ± 2.44	Hs00156548_m1	Protein binding
CSTA	4.58 ± 0.42	9.71 ± 2.05	Hs00193257_m1	Protease binding
DDIT3	27.43 ± 8.54	203.64 ± 20.65	Hs00358796_g1	Nucleic acid binding
DMRT1	6.68 ± 1.45	21.91 ± 3.11	Hs00232766_m1	Transcription factor
EGF	10.27 ± 4.08	3.52 ± 1.80	Hs01099999_m1	Signal transducer
EGR1	4.06 ± 1.67	87.38 ± 15.36	Hs00152928_m1	Transcription factor
FOXC1	1.96 ± 1.24	5.91 ± 3.68	Hs00559473_s1	Transcription factor
GCLM	3.48 ± 1.76	15.00 ± 9.08	Hs00157694_m1	Glutamate-cysteine ligase
GPNMB	4.75 ± 0.53	38.43 ± 6.90	Hs01095669_m1	Integrin binding
HMOX1	11.01 ± 2.30	26.19 ± 5.21	Hs01110251_m1	Heme oxygenase activity
ID2	4.48 ± 1.09	19.82 ± 4.24	Hs00747379_m1	Transcription repressor
JDP2	5.56 ± 2.14	24.51 ± 3.22	Hs00185689_m1	Transcription factor
LAMP3	6.08 ± 2.82	2.35 ± 1.09	Hs00180880_m1	Integral to membrane
LY96	2.14 ± 0.23	3.82 ± 2.42	Hs00209771_m1	Receptor activity
PEG3	21.38 ± 8.36	87.45 ± 30.33	Hs00377844_m1	Transcription factor
S100P	102.62 ± 31.88	570.45 ± 45.47	Hs00195584_m1	Calcium-dependent protein
SESN2	7.34 ± 4.98	23.62 ± 6.63	Hs00230241_m1	Cell cycle arrest
SPINK1	247.34 ± 23.27	465.02 ± 53.98	Hs00162154_m1	Endopeptidase inhibitor
SPRR1A	4.22 ± 4.49	70.57 ± 20.12	Hs00954595_s1	Structural molecule
TRIM16	2.08 ± 1.19	4.32 ± 2.56	Hs00414879_m1	DNA binding
TUBA1A	2.06 ± 1.27	3.46 ± 0.80	Hs00362387_m1	Structural molecule
WDR19	2.80 ± 1.32	9.43 ± 4.26	Hs00228414_m1	Transmembrane signaling

**Table 1b:** (b)

Gene	Fold downregulation 20 *μ*g/mL	Fold downregulation 40 *μ*g/mL	Assay ID	Function
ANKRD12	3.83 ± 1.84	21.63 ± 6.80	Hs00209530_m1	Ion channel inhibitor
ASPM	2.36 ± 0.63	5.69 ± 2.34	Hs00996458_m1	Calmodulin binding
CDC2	5.50 ± 2.22	9.39 ± 4.12	Hs00938777_m1	Protein binding
CDC25A	15.65 ± 3.26	2.09 ± 1.78	Hs00947994_m1	Phosphatase activity
CDCA4	7.98 ± 2.45	3.34 ± 1.85	Hs00743989_s1	Cell division
CENPE	78.27 ± 25.90	52.49 ± 16.37	Hs00156507_m1	Chromatin binding
CHEK1	24.20 ± 3.23	60.16 ± 29.80	Hs00176236_m1	Protein Kinase
COL12A1	3.62 ± 0.59	2.64 ± 1.23	Hs00329355_s1	Structural molecule
DLGAP5	12.34 ± 5.89	4.78 ± 2.63	Hs00207323_m1	Phosphatase activity
DPP4	15.39 ± 4.21	15.78 ± 2.62	Hs00175218_m1	Aminopeptidase
DTL	3.88 ± 1.40	1.55 ± 0.80	Hs00978565_m1	Protein binding
E2F8	75.55 ± 30.92	80.34 ± 19.30	Hs00226635_m1	Transcription factor
EBP	9.75 ± 3.21	3.26 ± 1.05	Hs00198130_m1	Sterol isomerase
FBXO5	4.25 ± 2.17	3.75 ± 0.54	Hs00559989_m1	Protein binding
FOLH1	5.92 ± 2.39	5.23 ± 1.31	Hs00379515_m1	Carboxypeptidase
HIST1H4C	8.34 ± 5.41	2.73 ± 0.73	Hs00543883_s1	DNA binding
HSP90B1	26.21 ± 12.03	30.88 ± 13.74	Hs00427665_g1	Nucleotide binding
ITGAV	13.50 ± 2.31	2.68 ± 1.86	Hs00233790_m1	Receptor
KIF14	1.93 ± 1.24	15.28 ± 4.89	Hs00208408_m1	Microtubule motor
MAD2L1	5.55 ± 3.25	7.83 ± 2.16	Hs01554515_g1	Protein binding
METAP2	5.04 ± 1.20	15.45 ± 5.21	Hs01127366_m1	Aminopeptidase
NRIP1	24.88 ± 6.20	45.47 ± 14.54	Hs00942766_s1	Transcription coactivator
POLA1	15.62 ± 3.22	4.39 ± 2.87	Hs00213524_m1	Nucleotide binding
PRIM2	11.83 ± 5.41	2.32 ± 0.33	Hs00386277_m1	DNA primase
RRM1	5.87 ± 1.72	11.23 ± 4.21	Hs01040698_m1	Ribonucleoside diphosphate reductase
SKP2	17.52 ± 11.90	16.43 ± 4.23	Hs00180634_m1	Ubiquitin-protein ligase
TOP2A	15.53 ± 3.89	10.34 ± 2.11	Hs01032127_g1	Topoisomerase activity
TOP2B	2.03 ± 1.48	2.25 ± 0.79	Hs00172259_m1	Topoisomerase activity
TPR	1.35 ± 1.91	4.66 ± 1.30	Hs00162918_m1	Protein kinase
UHRF1	10.73 ± 2.84	3.41 ± 1.52	Hs00273589_m1	Transcription factor

## Abbreviations

EENL: Ethanol extract of neem leaves
ATF3: Activating transcription factor 3
EGR1: Early growth response-1
HMOX1: Heme oxygenase-1
VEGF: Vascular endothelial growth factor
TEM: Transmission electron microscopy.
